# Metabolite Profiling of Four Major Flavonoids of *Herba Epimdii* in Zebrafish

**DOI:** 10.3390/molecules17010420

**Published:** 2012-01-04

**Authors:** Yingjie Wei, Ping Li, Hongwei Fan, E Sun, Changmei Wang, Luan Shu, Wei Liu, Xiaolu Xue, Qian Qian, Xiaobin Jia

**Affiliations:** 1 Key Laboratory of New Drug Delivery System of Chinese Materia Medica, Jiangsu Provincial Academy of Chinese Medicine, 100 Shizi Street, Nanjing 210028, China; Email: sune0825@yahoo.com.cn (E.S.); hanxi186@yahoo.com.cn (C.W.); shuluan2006@hotmail.com (L.S.); sophia.liuwei@qq.com (W.L.); xl.jow@163.com (X.X.); qq369461394@163.com (Q.Q.); 2 State Key Laboratory of Natural Medicines and Department of Pharmacognosy, China Pharmaceutical University, 24 Tongjia Lane, Nanjing 210009, China; Email: liping2004@126.com; 3 Lab of Clinical Pharmacology, Nanjing Medical University, Affiliated Nanjing First Hospital, 68 Changle Road, Nanjing 210006, China; Email: fanhongwei178@sina.com

**Keywords:** zebrafish, icariin, baohuoside I, epimedin A, epimedin C, *Herba Epimedii*, metabolism

## Abstract

The zebrafish model organism was applied first in a metabolic study of icariin, baohuoside I, epimedin A and epimedin C, which are flavonoids in *Herba Epimedii*. Metabolites of these compounds in zebrafish after exposure for 24 h were identified by HPLC-ESI-MS, whereby the separation was performed with a Zorbax C-18 column using a gradient elution of 0.05% formic acid acetonitrile-0.05% formic acid water. The quasi-molecular ions of compounds were detected in simultaneous negative and positive ionization modes. Metabolic products of icariin and epimedin C via cleavage of glucose residue instead of rhamnose residues were found, which coincided with the results using regular metabolic analysis methods. In addition, the zebrafish model was used to predict the metabolism of the trace component epimedin A, whose metabolic mechanisms haven’t been clearly elucidated with the current metabolism model. The metabolic pathway of epimedin A in zebrafish was similar to those of its homologue icariin and epimedin C. Our study demonstrated that the zebrafish model can successfully imitate the current models in elucidating metabolic pathways of model flavonoids, which has advantages of lower cost, far less amount of compound needed, easy set up and high performance. This novel model can also be applied in quickly predicting the metabolism of Chinese herb components, especially trace compounds.

## 1. Introduction

The material basis of Chinese herbs is the essence of research in Traditional Chinese Medicines (TCMs). It is considered that the real therapeutic effects of TCMs may depend on the transformative components after metabolism *in vivo* rather than prototype components. Metabolic study of TCMs is crucial and has been applied effectively in the major fields of TCMs research, including material basis, mechanism of action and quality control, *etc.* However, there are still limitations for metabolic study methods so far using either *in vivo* or *in vitro* models in TCM research as we described before [1]. Therefore, it is of great importance to develop an alternative metabolic study method combining the advantages of current methods and overcome shortages to make it available for even trace compounds analysis. Based on genetic and physiological advantages of zebrafish, we have put forward for the first time a method for the metabolic study of TCMs using a zebrafish model [1,2,3]. 

In our previous study, a zebrafish model was successfully established and applied in a metabolism study of three typical model saponins of Sanqi, where their metabolites by deglycosylation and hydroxylation in zebrafish coincided with other reports using regular metabolic methods, which suggested that the zebrafish model could be a prospectively useful metabolic method for predicting the metabolism of trace compounds from herbal medicines [1]. However, here other important and interesting issues to be investigated are whether or not this model is valuable in profiling metabolites of other typical types of compounds from TCM, and whether or not this model can be used in predicting metabolites profiles of compounds whose metabolic mechanisms have not been elucidated clearly yet therefore, it is essential to carry out further metabolic experiments using zebrafish. 

**Figure 1:**
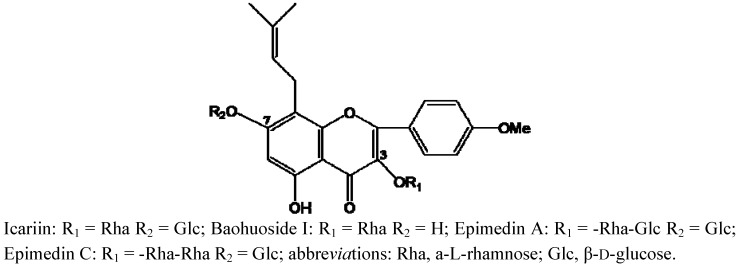
Structures of icariin, baohuoside I, epimedin A and epimedin C in *Herba Epimedii* in this study.

In addition to saponins, flavonoids are also very important type of compounds in Chinese herbs. Here four typical flavonoids—Icariin, baohuoside I, epimedin A and epimedin C—from a well known Chinese medicine named *Herba Epimdii* (Yingyanghuo in Chinese, YYH) were selected as model flavonoids. These flavonoids were selected because they are usually considered as the main pharmacological components of YYH for quality control, pharmacokinetic and metabolic research purposes [4,5,6,7,8,9,10,11,12,13,14,15,16,17,18,19,20,21]. Previous investigations presumed that the major metabolic pathway of icariin and epimedin C is hydrolysis of 7-O glucosides, while baohuoside I can hard cleave its rhamnose residue [12,13,16,17,18]. Since it is difficult to get enough amounts of compounds from YYH, there are few research reports so far about the *in vivo* metabolism of other flavonoids in YYH such as epimedin A. However, the alternative model using zebrafish may be helpful for metabolic studies of such trace compounds. In the present study, zebrafish was used in metabolism study of icariin, baohuoside I and epimedin C (Figure 1), whose metabolic mechanisms have been clearly elucidated, to see if zebrafish can imitate current methods in elucidating the metabolism of types of compounds other than saponins, and zebrafish was also used in a metabolism study of their homologue epimedin A (Figure 1), whose metabolic mechanisms has not been clarified fully, so see if zebrafish can be used in predicting the metabolism of trace compound, in addition to previous reports using zebrafish larvae [1,22], our results provide update and valuable evidence of the feasibility and reasonability of the novel model using adult zebrafish in the metabolic study of trace components of TCMs.

## 2. Results and Discussion

### 2.1. Analysis of the Metabolic Components of Icariin, Baohuoside I, Epimedin A and Epimedin C after Zebrafish Exposure by HPLC-ESI-MS

Icariin, baohuoside I, epimedin A, epimedin C and their metabolites after zebrafish exposure were identified by HPLC-ESI-MS with an ESI source in simultaneous negative and positive modes, which gave complementary MS data information. The responses of these components in negative mode were more sensitive than in positive mode, and quasi-molecular ions of [M−H]^−^, [2M−H]^−^, [M+HCOO]^−^, [M+H]^+^ and [M+Na]^+^ were used for molecule mass information. By attentive study of the mass spectra of compounds and comparison with reference data and some standards, deglycosylation metabolites of icariin, epimedin A and epimedin C but not baohuoside I were identified by comparison with blank samples. 

**Figure 2:**
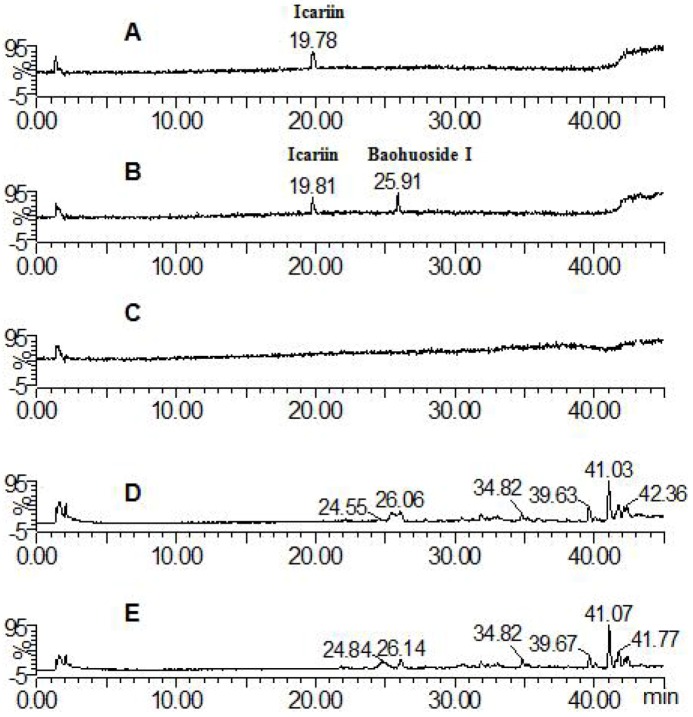
Mass spectrometry total ion current chromatograph for the icariin groups after 24 h (negative mode). **A**: icariin solution; **B**: icariin zebrafish solution; **C**: blank zebrafish group solution; **D**: zebrafish of icariin zebrafish group; **E**: zebrafish of blank zebrafish group.

The parent component icariin and its transformative metabolite baohuoside I were found in both solution and zebrafish of the icariin group (total ion current chromatographs are shown in Figure 2). One peak (t_R_: 19.81 min) exhibited quasi-molecular ions of [M−H]^−^675.26, [M+HCOO]^−^721.70, [M+H]^+^677.88 and [M+Na]^+^699.58, hence the MW was deduced as 676. By comparison with a standard the peak was identified as icariin. The other peak (t_R_: 25.91 min) exhibited quasi-molecular ions of [M−H]^−^513.91, [2M−H]^−^1027.61, [M+H]^+^515.83 and [M+Na]^+^537.81, hence the MW was deduced as 514, or 162 Da less than that of icariin; by comparison with a standard, the peak was identified as baohuoside I, a degradation product derived from icariin via cleavage of a glucose residue on the 7-OH of ring A. These results are consistent with previous reports of rat metabolism *in vivo* and rat intestinal bacteria metabolism *in vitro* [12,13].

**Figure 3:**
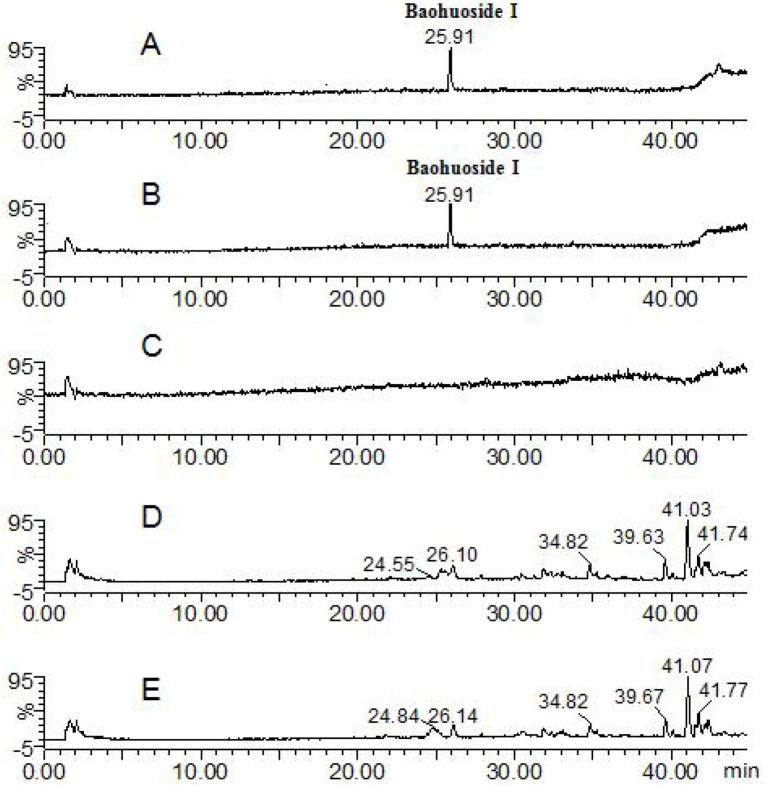
Mass spectrometry total ion current chromatograph for the baohuoside I groups at 24 h (negative mode). **A**: baohuoside I solution; **B**: baohuoside I zebrafish solution; **C**: blank zebrafish solution; **D**: zebrafish of baohuoside I zebrafish; **E**: zebrafish of blank zebrafish.

Except for the parent component baohuoside I, no other transformative metabolite was found in both solution and fish of the baohuoside I zebrafish group (total ion current chromatographs are shown in Figure 3). The peak with t_R_: 25.91 min exhibited quasi-molecular ions of [M−H]^−^513.91, [2M−H]^−^1027.61, [M+H]^+^515.76 and [M+Na]^+^537.88, hence the MW was deduced as 514. By comparison with a standard this peak was identified as the parent component baohuoside I. The result is consistent with previous reports on the rat intestinal bacteria metabolism *in vitro* [13].

**Figure 4:**
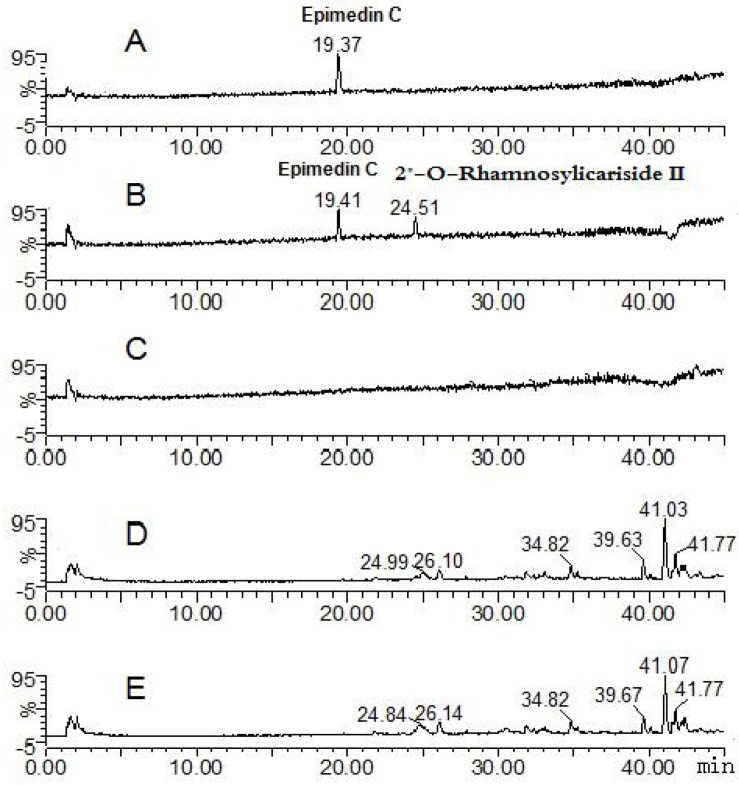
Mass spectrometry total ion current chromatograph for the epimedin C groups after 24 h (negative mode). **A**: epimedin C solution; **B**: epimedin C zebrafish solution; **C**: blank zebrafish group solution; **D**: zebrafish of epimedin C zebrafish group; **E**: zebrafish of blank zebrafish group.

The parent component epimedin C and its transformative metabolite 2"*-O-*rhamnosylicariside II were found in solution of the epimedin C zebrafish group (total ion current chromatographs are shown in Figure 4). One peak (t_R_: 19.41 min) exhibited quasi-molecular ions of [M−H]^−^821.79, [M+HCOO]^−^867.68, [M+H]^+^823.71 and [M+Na]^+^845.49, hence the MW was deduced as 822, in addition, *m/z* 659.74 [M−H−162]^−^, 677.81 [M+H−146]^+^ and 531.84 [M+H−162−146 + 16]^+^ were characteristic fragment ions of epimedin C formed via loss of the glucose, rhamnose and both glucose and rhamnose residues, respectively. By comparison with a standard, the peak was identified as epimedin C. The other peak (t_R_: 24.51 min) exhibited quasi-molecular ions of [M−H]^−^659.74, [M+H]^+^661.87 and [M+Na]^+^683.71, hence the MW was deduced as 660, or 162 Da less than that of epimedin C; in addition, *m/z* 515.90 [M+H−146]^+^ was a characteristic fragment ion of 2"*-O-*rhamnosylicariside II, formed via loss of a rhamnose residue. By further referring to the literature [13,16,17,18], the t_R_: 24.51 min peak was identified as 2"-*O*-rhamnosylicariside II, a degradation product derived from epimedin C via cleavage of one glucose residue on the 7-OH of ring A. The result is consistent with that of perfused rat intestinal and rat *in vivo* models [13,16].

**Figure 5:**
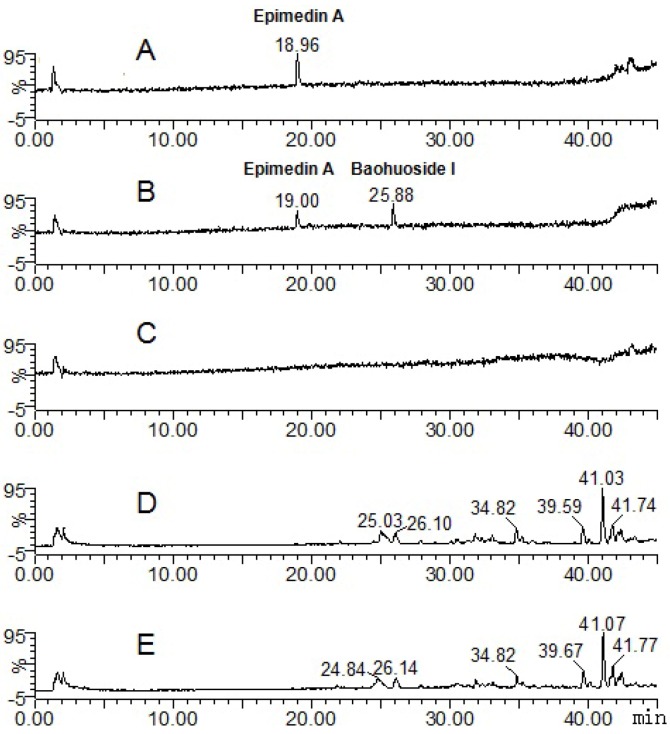
Mass spectrometry total ion current chromatograph for the epimedin A groups after 24 h (negative mode). **A**: epimedin A solution; **B**: epimedin A zebrafish group solution; **C**: blank zebrafish group solution; **D**: zebrafish of epimedin A zebrafish group; **E**: zebrafish of the blank zebrafish group.

The parent component epimedin A and its transformative metabolite baohuoside I were found in the epimedin A zebrafish group solution (total ion current chromatographs are shown in Figure 5). One peak (t_R_: 19.00 min) exhibited quasi-molecular ions of [M−H]^−^837.73, [M+HCOO]^−^883.62, [M+H]^+^839.72 and [M+Na]^+^861.84, hence the MW was deduced as 838, in addition, *m/z* 675.81 [M−H−162]^−^, 677.74 [M+H−162]^+^ and 531.84 [M+H−162−162 + 16]^+^ were characteristic fragment ions of epimedin A, formed via loss of one or two glucose residues. By comparison with a standard, the peak was identified as epimedin A. Another peak (t_R_: 25.88 min) exhibited quasi-molecular ions of [M−H]^−^513.84, [2M−H]^−^1027.74, [M+H]^+^ 515.76 and [M+Na]^+^537.88, hence the MW was deduced as 514, or 324 Da less than that of epimedin A. By comparison with a standard this peak was identified as baohuoside I, a degradation product derived from epimedin A via cleavage of two glucose residues on both the 7-OH of ring A and the 3-OH of ring D. 

**Figure 6:**
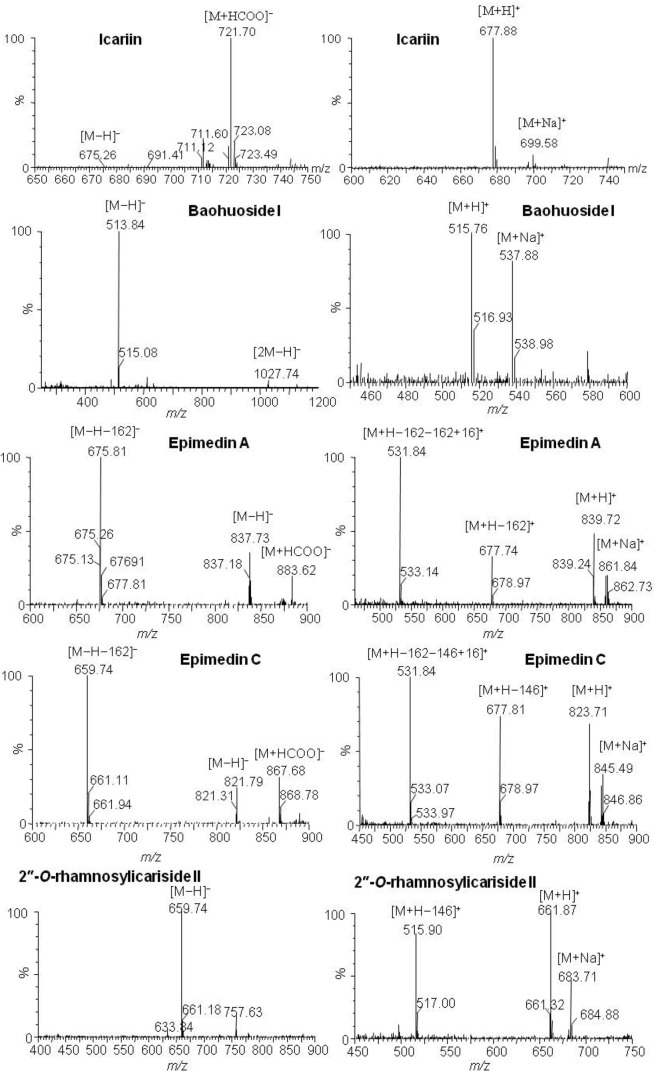
Representative MS spectra of icariin, baohuoside I, epimedin A, epimedin C and their transformative components by zebrafish.

**Figure 7:**
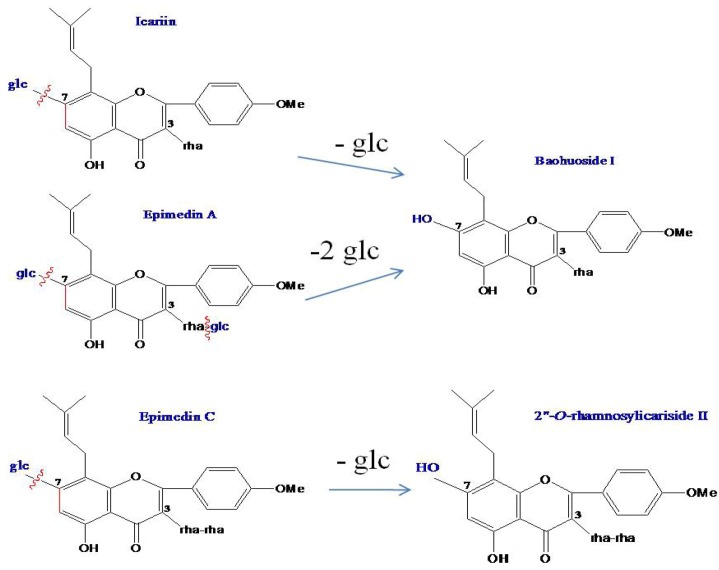
The possible metabolic pathways of icariin, epimedin A and epimedin C by zebrafish.

MS data for icariin, baohuoside I, epimedin A, epimedin C and the metabolites produced by zebrafish are shown in Table 1, the representative MS spectra are shown in Figure 6, and the possible metabolic pathways of icariin, epimedin A and epimedin C are elucidated in Figure 7.

**Table 1:** MS data for icariin, baohuoside I, epimedin A, epimedin C and their metabolites after zebrafish exposure for 24 h.

**Compounds**	**Retention time (min)**	**quasi-molecular ions **				**MW**	**Metabolite presumed**	**Zebrafish**		**Current metabolism (references)**
		[M−H]^−^	[M+HCOO]^−^	[M+H]^+^	[M+Na]^+^			**solution**	**body**	
Icariin	19.81	675.26	721.70	677.88	699.58	676	Icariin	+	+	
	25.91	513.91		515.83	537.81	514	Baohuoside I	+	+	[10,11]
Baohuoside I	25.91	513.84		515.76	537.88	514	Baohuoside I	+	+	[11]
Epimedin A	19.00	837.73	883.62	839.72	861.84	838	Epimedin A	+		
	25.88	513.84		515.76	537.88	514	Baohuoside I	+		
Epimedin C	19.41	821.79	867.68	823.71	845.49	822	Epimedin C	+		
	24.51	659.74		661.87	683.71	660	2" *-O-*rhamnosylicariside II	+		[13]

+ detected.

### 2.2. Rationality and Advantages of Metabolic Study with Zebrafish Compared to the Existing Model

As for icariin, baohuoside I and epimedin C, whose metabolic mechanisms have been clearly elucidated, their metabolic pathways in zebrafish are highly consistent with the existing metabolism results. Over the past ten years, many *in vivo* or *in vitro* metabolic studies of icariin [11,12,13,14,15,16,17] and epimedin C [15,16] have been reported. Their common mechanism were elucidated to be that icariin and epimedin C are both metabolized *in vivo* via cleavage of glucose residues into baohuoside I and 2"-*O*-rhamnosylicariside II, respectively, but it is difficult for their metabolites to further degrade rhamnose residues. Our present study found for the first time the same metabolic mechanism of icariin, baohuoside I and epimedin C in the zebrafish model as in the current model, which provides powerful evidence for the rationality of zebrafish model.

As for epimedin A, whose metabolic mechanisms have not been clarified fully, its metabolic mechanism was successfully predicted by the zebrafish model. To date, except for a report using a perfused rat intestinal model [15], no other regular *in vivo* and *in vitro* metabolism of epimedin A was reported. In that study it was found that the main metabolite of epimedin A was the degradation product named sagittatoside A, resulting from cleavage of one glucose residue [15]. In the present study using zebrafish, epimedin A was transformed into baohuoside I instead of sagittatoside A via cleavage of both 7-OH and 3-OH glucose residues, and no metabolite resulting from cleavage of the rhamnose residue was found. The result matched the metabolic mechanism of the homologous compounds icariin and epimedin C, which suggested that the metabolisms of epimedin A in zebrafish were rational. 

Conditions selected for zebrafish metabolism experiments were similar to those of our previous study [1], which were simple, reasonable and feasible with much lower labor intensity and lower cost. In addition, only small amounts of icariin, baohuoside I, epimedin A and epimedin C (1.16, 1.3, 1.18 and 1.57 mg, respectively) were required, far less than those needed in rat metabolism studies, which make it possible to predict the metabolism of large numbers of trace components *in vivo*. 

## 3. Experimental

### 3.1. Chemicals and Reagents

Icariin (purity >98%) was purchased from the National Institute for the Control of Pharmaceutical and Biological Products (Beijing, China), while baohuoside I, epimedin A and epimedin C (all >98% purity) were provided by the Laboratory of Pharmaceutical Preparation (Jiangsu Provincial Academy of Chinese Medicine, China). HPLC grade acetonitrile was purchased from Tedia Company (Fairfield, CT, USA), deionized water was purified by Milli-Q system (Millipore, Bedford, MA, USA), robust purified water, physiological saline (sodium chloride injection) were from Nanjing Xiaoying Pharmaceutical Group Co. Ltd. (Nanjing, China), dimethyl sulfoxide (DMSO) from Sinopharm Chemical Reagent Co. Ltd (Shanghai, China), and the other reagents were of analytical grade.

### 3.2. Animals

The adult zebrafish (*D. rerio*) of mixed sex were provided by Model Animal Research Center of Nanjing University (Nanjing, China), and acclimatized to tap water in a glass aquarium for at least 10 days prior to experimentation. Fish were kept at a temperature of 25 ± 1 °C in a photoperiod of 12:12 h. The fish were fed daily during the acclimatization period, and were fasted overnight before the day of the experiment.

### 3.3. Instruments

A Waters Alliance 2695-ZQ 2000 HPLC-MS system 2695 liquid chromatography system (Waters Corporation, Milford, MA, USA) consisting of a quadruple pump, an autosampler, column temperature controller and a PDA detector, Micromass ZQ2000 single-quadrupole mass spectrometer (Waters) with an electrospray ionization source, Masslynx 4.0 ChemStation software; Mettler Toledo AB135-S Analytical Balance (Mettler Toledo, Schwerzenbach, Switzerland); KQ3200DE Digital Ultrasonic Washer (Kunshan Ultrasonic Instruments Co. Ltd, Kunshan, China); Labconco Freezer Dryer (Labconco, kansas, MO, USA); TGL-16G Desk Centrifuge (Shanghai Anting Scientific Instrument Factory, Shanghai, China), Organomation N-EVAP^TM^ 112 Nitrogen Evaporator (Organomation Associates, Inc., Berlin, MA, USA).

### 3.4. Biological Sample Collection

Adult zebrafish were divided into five experimental groups of five fish each after fasting for 12 h, one blank control group was exposed to 1% DMSO purified water (blank zebrafish group), four groups were exposed to 30 mL solution of icariin (5.8 µg/mL), baohuoside I (6.5 µg/mL), epimedin A (7.0 µg/mL) and epimedin C (7.85 µg/mL) in 1% DMSO purified water (drug zebrafish groups), respectively. In addition, the above solutions of icariin (5.8 µg/mL), baohuoside I (6.5 µg/mL), epimedin A (7.0 µg/mL) and epimedin C (7.85 µg/mL) without zebrafish were used as blank drug controls. Both zebrafish body and solution of the blank zebrafish group and drug zebrafish groups were sampled at 24 h, respectively. the zebrafish bodys of each group were combined and washed rapidly with 1% DMSO purified water three times and stored at −70 °C prior to analysis; the solution of each group were also combined, respectively, then 8 mL (n = 3) combined solution of each group were sampled and also stored at −70 °C prior to analysis. Solution of blank drug control group were sampled as above (8 mL, n = 3) at 0 h, 24 h.

### 3.5. Sample Preparation

The solution sample (8 mL) was freeze-dried to dryness, and the residue was dissolved in 90% methanol (1 mL). After centrifugation at 15,000 rpm for 15 min, supernatant (20 μL) was introduced into the HPLC-MS system for analysis. The zebrafish body samples (five fish of each group) were cut with scissors, and 1 g was sampled and homogenized with physiological saline (5 mL), followed by centrifugation at 3,500 rpm for 15 min, then the supernatant was vortex mixed with methanol (20 mL), after centrifugation at 3,500 rpm for 15 min, the supernatant was evaporated to dryness with nitrogen at 40 °C, and the residue was dissolved in 90% methanol (1 mL). After centrifugation at 15,000 rpm for 10 min, 20 μL of the supernatant was injected into the HP LC-MS system for analysis.

### 3.6. Analysis Condition

HPLC-MS was performed with a Waters Alliance 2695—ZQ 2000 single—quadrupole mass spectrometer equipped with an electrospray ionization source. The HPLC analysis was carried out on the column configuration consisted of an Agilent Zorbax Extend reversed - phase C_18_ column (5 μm, 150 mm × 4.6 mm) and an Agilent Zorbax extend—C_18_ guard column (5 μm, 4.6 mm × 12.5 mm). The column was maintained at 30 °C, the flow rate was 1.0 mL/min. A gradient elution of 0.05% aqueous formic acid (A) and 0.05% acetonitrile formic acid (B) was used as 10% B at 0–5 min, 10–25% B at 5–15 min, 25–90% B at 15–40 min, 90%B at 40–45 min. The mass spectra were recorded in simultaneous positive and negative ionization full-scan mode throughout the *m/z* range 100~1,200, capillary voltage 3.0 kV, cone voltage 30 V, drying gas flow rate 320 L/h, ion source temperature 120 °C, adjuvant gas temperature 310 °C, extract ions current (EIC): [M−H]^−^; [2M−H]^−^; [M+HCOO]^−^; [M+H]^+^; [M+Na]^+^.

## 4. Conclusions

Based on our previous metabolic study of saponins in a zebrafish model, in the present report we further demonstrated the feasibility of the zebrafish model as a useful one in the metabolic study of components in Chinese herbs, especially for those like flavonoids found in micro amounts, such as icariin, baohuoside I, epimedin A and epimedin C from YYH. The results showed that the zebrafish model could imitate the current metabolic model successfully in elucidating metabolic mechanisms of icariin, baohuoside I and epimedin C. Moreover, the proposed zebrafish model also greatly helped to predict metabolism of epimedin A, whose metabolic mechanisms hasn’t been clearly clarified by the current models. Our results confirmed the hypothesis that this novel, powerful model can be applied to quickly predicating *in vivo* drug metabolism, especially for those trace compounds of TCMs, with the advantages of lower cost, easier set up and higher performance.
